# Relaxant Effect of the Ethanol Extract of *Helichrysum plicatum* (Asteraceae) on Isolated Rat Ileum Contractions 

**DOI:** 10.3390/molecules15053391

**Published:** 2010-05-10

**Authors:** Dubravka Bigovic, Suzana Brankovic, Dusanka Kitic, Mirjana Radenkovic, Teodora Jankovic, Katarina Savikin, Slavoljub Zivanovic

**Affiliations:** 1 Institute for Medicinal Plants Research, Tadeuša Košćuška 1, 11000 Belgrade, Serbia; E-Mails: dbigovic@mocbilja.rs (D.B.); tjankovic@mocbilja.rs (T.J.); ksavikin@mocbilja.rs (K.S.); 2 Department of Physiology, Faculty of Medicine, University of Nis, Bulevar dr Zorana Djindjica 81, 18000 Nis, Serbia; E-Mails: brankovic.suzana@yahoo.com (S.B.); mirjanakos@medfak.ni.ac.rs (M.R.); 3 Department of Pharmacology, Faculty of Medicine, University of Nis, Bulevar dr Zorana Djindjica 81, 18000 Nis, Serbia; E-Mail: szivanovic@medfak.ni.ac.rs (S.Z.)

**Keywords:** spasmolytic, *Helichrysum plicatum*, ileum, rats

## Abstract

*Helichrysum plicatum* (Turkish Helichrysum) has been used in folk medicine for the treatment of gastric and hepatic disorders. The aim of the present study was to examine the relaxant activity of an extract of *H. plicatum* flowers on isolated rat ileum. Segments of ileum of rats were suspended in an organ bath. Cumulative concentrations of *H. plicatum* ethanol extract induced a relaxant effect on spontaneous rat ileum contractions. *H. plicatum* extract caused a mean contractile response of 81.68 ± 6.17% (at a dose of 0.01 mg/mL) and 30.08 ± 9.07% (at a dose of 1 mg/mL). A similar effect was observed with papaverine (0.01–3 μg/mL). *H. plicatum* extract (0.01–1 mg/mL) relaxed high K^+^ (80 mM) precontractions, an effect similar to that caused by papaverine (0.01–3 μg/mL). The plant extract (0.03–0.3 mg/mL) also induced a significant depression of the cumulative concentration response curve for acetylcholine (5–1500 nM) (p < 0.01). Atropine (140 nM) abolished the acetylcholine effect. The extract (0.03–0.3 mg/mL) reduced the histamine (1–300 nM) and BaCl_2_ (3–900 μM) induced contractions (p < 0.01). Our results showed the relaxant effect of the ethanol extract of *Helichrysum plicatum* flowers on the isolated rat intestine Extract of *H. plicatum* can inhibit the spontaneous ileum contractions and contractions induced by acetylcholine, histamine, barium and potassium ions.

## 1. Introduction

Plants are sources of many medicinal drugs. For centuries, herbs have been used in traditional medicine to treat of many gastrointestinal disorders. In recent times numerous scientific studies have been performed to test the potential effect of plant extracts on intestinal contractions [1]. However, the mechanism of action by which these plants exert their therapeutic effects has not been completely elucidated [2].

The genus *Helichrysum* (Asteraceae) comprises approximately 500-600 species. Species from the genus *Helichrysum* are used in Europe and Africa in the treatment of various medical conditions [3]. *Helichrysum* spp. have been used in folk medicine for at least 2,000 years as choleretics, cholagogues, hepatoprotectives and for stimulation of the secretion of gastric juice [4,5]. Some members of the genus are known for their anti-inflammatory, anti-allergic and antibacterial properties [6]. In Turkish folk medicine *H. plicatum* has been used as a diuretic, lithagogue, and for stomach ache [7]. *H. plicatum* has been used in Serbian and Macedonian folk medicine for the treatment of gastric and hepatic disorders. Moreover, the antidiabetic, and antioxidant activity of this species have also been reported [8]. 

The aim of the present study was to evaluate the possible spasmolytic activity of the ethanol extract of *Helicrisum plicatum* on rat ileum, since there is no data of the physiological effects of the extract on isolated rat intestine. 

## 2. Results and Discussion

**Figure 1:**
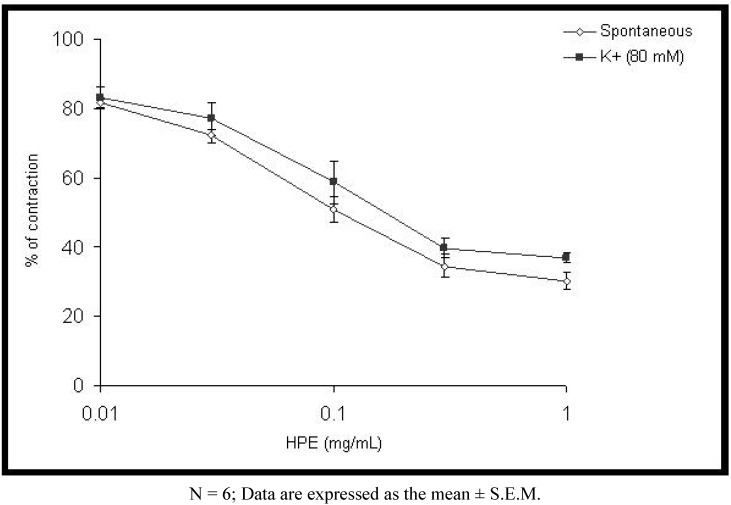
Inhibitory effect of the ethanol extract of *Helichrysum plicatum* (HPE) on spontaneous and high K^+^-induced contractions in isolated rat ileum.

**Figure 2:**
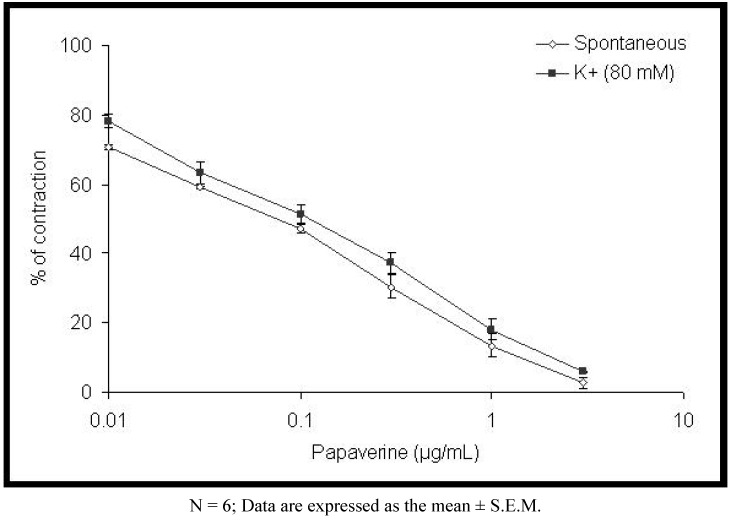
Inhibitory effect of papaverine on spontaneous and high K^+^-induced contractions in isolated rat ileum.

The results presented in Figure 1 demonstrate that *H. plicatum* extract in cumulative concentrations (0.01–1 mg/mL) induced a concentration-dependent inhibition of the spontaneous contractions of the rat ileum. Extract of *H. plicatum* caused a mean contractile response of 81.68 ± 6.17% (at a concentration of 0.01 mg/mL) and 30.08 ± 9.07%, at a concentration of 1 mg/mL (p < 0.01). The calculated EC_50_ value was 0.13 ± 0.01 mg/mL. A similar effect was observed with papaverine (EC_50_ value was 0.06 ± 0.003 μg/mL, Figure 2). 

The extract of *H. plicatum* (0.01–1 mg/mL) relaxed the tonic contraction induced by KCl in a concentration dependent manner, with an EC_50_ value of 0.22 ± 0.03 mg/mL (Figure 1). Papaverine (EC_50_ 0.09 ± 0.008 μg/mL) was also used as a positive control. Acetylcholine caused a concentration dependent contraction of rat ileum. Plant extract (0.03–0.3 mg/mL) induced a significant depression of the cumulative concentration response curve for acetylcholine (p < 0.01). Extract of *H. plicatum* caused a modification of the EC_50_ of acetylcholine from 58.86 ± 4.58 nM (in the absence of the extract) to 2,624.24 ± 104.69 nM, in the presence of the extract at a concentration of 0.3 mg/mL (Table 1). 

**Table 1:** EC_50_ and E_max_ values obtained from the cumulative dose-response curves to acetylcholine.

Antagonist	EC_50_ (nM) mean±S.E.M	E_max_ %±S.E.M.
Control	58.86 ± 4.58	100
*Helichrysum plicatum* (mg/mL)		
0.03	951.28 ± 85.74^**^	49.87 ± 5.56^**^
0.1	1,587.25 ± 95.42^**^	34.48 ± 4.21^**^
0.3	2624.24 ± 104.69^**^	14.79 ± 4.22^**^
Atropine (140 nM)	13,984.61 ± 967.25^***^	6.25 ± 0.58^***^

S.E.M.: standard error of the mean; ^**^p < 0.01 and ^***^ p < 0.001.

**Figure 3:**
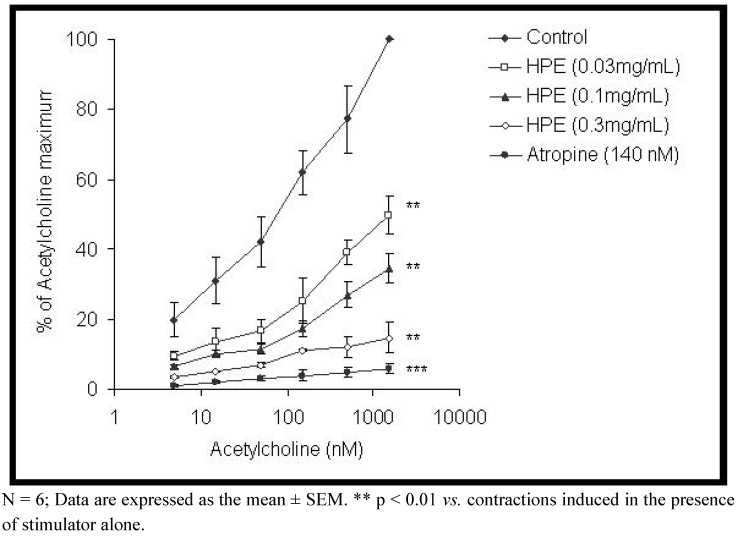
Comparison of dose-response curves of acetylcholine in absence and presence of the ethanol extract of *Helichrysum plicatum* (HPE) and atropine in isolated rat ileum.

Pretreatment of the tissue with atropine (140 nM) abolished the acetylcholine effect (Figure 3). 

**Figure 4:**
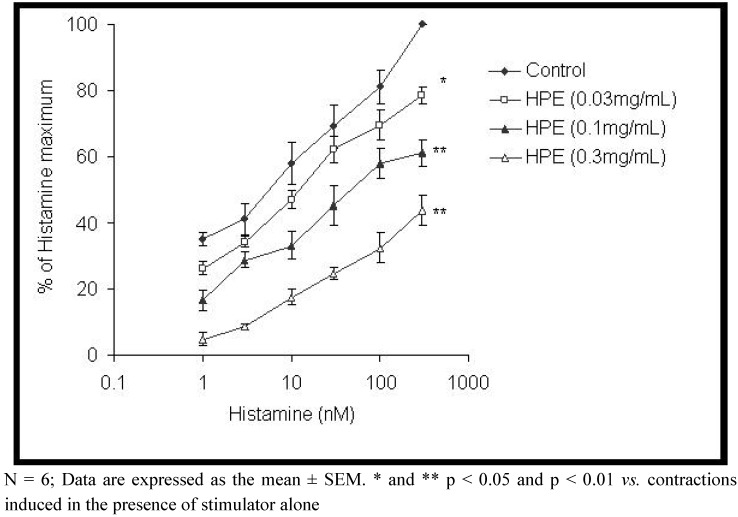
Comparison of dose-response curves of histamine in absence and presence of *Helichrysum plicatum* extract (HPE) in isolated rat ileum.

Histamine was used as agonist for stimulating ileum smooth muscle. The extract of *H. plicatum* significantly inhibited the histamine induced contractions in a concentration dependent manner (p < 0.01), with maximal inhibition of 65.97 ± 6.90% (Figure 4). The EC_50_ values of the histamine (56.39 ± 6.35 nM) were modified by the extract of *H. plicatum* (EC_50_ of 1,044.34 ± 91.36 nM). 

**Figure 5:**
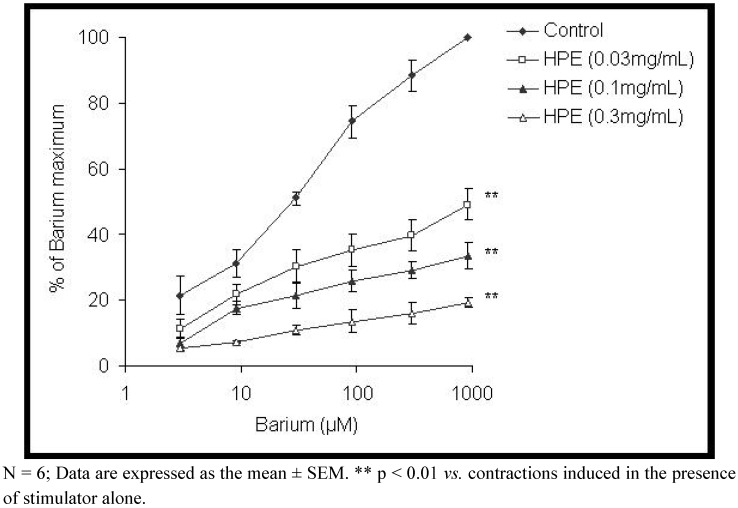
Comparison of dose-response curves of barium ions in absence and presence of *Helichrysum plicatum* extract (HPE) in isolated rat ileum.

The effects of the *H. plicatum* extract on BaCl_2_ induced contractions are shown in Figure 5. The extract of *H. plicatum* significantly reduced the BaCl_2_ contraction response from 50.94 ± 4.78% to 89.65 ± 9.02% (p < 0.01). The EC_50_ values of the BaCl_2_ (24.42 ± 9.58 μM) were affected by the extract of *H. plicatum* (EC_50_ of 1,096.63 ± 79.25 μM). 

The extract of *H. plicatum* was studied for its possible spasmolytic effect on rat intestine. Isolated ileum suspended in an organ bath showed spontaneous contractile activity. Our *in vitro* studies indicate that the plant extract produces relaxant effects on rat ileum spontaneous contractions. The effect was concentration-dependent and reversible after washing the tissue with Tyrode`s solution. The observed spasmolytic activity of *H. plicatum* extract was less potent than that of papaverine, a nonspecific smooth muscle relaxant. Papaverine is a dual inhibitor of Ca^2+^ influx [9] and phosphodiesterase inhibitor [10,11,12,13].

The enteric nervous system is considered to be an independent nervous system that controls and coordinates gastrointestinal motility. Gastrointestinal motility is regulated by numerous mediators, mainly acetylcholine, histamine, 5-hydroxytryptamine, bradykinins, prostaglandins, substance P and cholecistokinins which achieve their contractile effects through an increase in cytosolic Ca^2+ ^[14,15]. To clarify the possible underlying mechanism, we investigated the influence of the extract on acetylcholine, histamine, BaCl_2 _and KCl-induced smooth muscle contraction. 

It was found that the extract of *H. plicatum* exhibited a significant depressive effect on the cumulative concentration response curves for acetylcholine in the isolated rat ileum. Pretreatment with atropine abolished the contractile effect of acetylcholine. Acetylcholine, a neurotransmitter, is released by the parasympathetic nervous system, and plays an important physiological role in the regulation of gut movements [16]. The acetylcholine induced contractions of the rat ileum involve two different mechanisms coupled to muscarinic receptors. One mechanism activates non-selective cation channels in the plasma membrane, which results in membrane depolarization. The depolarization stimulates Ca^2+^ influx through voltage-gated Ca^2+^ channels. The other mechanism activates contraction by the release of intracellular calcium [17]. 

One of the possible mechanisms for the spasmolytic activity of the extract could be mediated through the inhibition of histaminic receptors. In our study the extract of *H. plicatum* inhibited the contractions of rat ileum exerted by histamine in a concentration dependent manner. Histamine is known to cause contraction of smooth muscle of the gastrointestinal tract. Application of histamine to intact smooth muscle produced a concentration-dependent membrane depolarization and increasedexcitability [18,19]. 

To specify the spasmolytic activity of *H. plicatum* extract, we tested its effect on the contraction of isolated rat ileum induced by BaCl_2_ and KCl, to find out if *H. plicatum* is a musculotropic substance. Barium ion (Ba^2+^) has been known to induce contractions in smooth muscle tissues. Ba^2+^ depolarizes the smooth muscle membrane and opens the voltage-dependent Ca^2+^ channels resulting in a Ca^2+^ influx [20]. *H. plicatum* extract produced a statistically significantly inhibition of the the contractions induced by BaCl_2_.

According to the literature a lot of plant secondary metabolites can mediate spasmolytic effects through inhibition of the influx of calcium into the cell. High K^+^ stimulation, which provokes membrane depolarization, is the most common method for the introduction of Ca^2+^ into cell without receptor stimulation [21]. High concentration of K^+^ cause smooth muscle contractions through opening voltage-dependent Ca^2+^ channels, thus allowing an influx of extracellular Ca^2+^ causing a contractile effect [22]. A substance causing inhibition of high K^+^- induced contractions is considered as a blocker of calcium influx [23]. 

Karaki *et al*. [20] have researched contractions of intestinal smooth muscle induced by Ba^2+^ and high concentrations of K^+^. They concluded that both contractions are due to influx of Ca^2+^. The extract of *H. plicatum* exhibited an inhibitory effect on intestine contractions induced by BaCl_2_ and high concentrations of K^+^. According to Gilani *et al*. [24] different plant extracts usually mediate their spasmolytic action through blockage of Ca^2+ ^influx. This indicates that the plant extract is preventing the increase of calcium ions and muscle tension [25,26,27]. The extract of *H. plicatum* caused relaxation of the K^+^-induced contractions, suggesting that the spasmolytic effect is possibly mediated through calcium channel blockage.

There is growing evidence that the spasmolytic effect of the extract is associated with the presence of phenolic compounds. Flavonoids are one of the most numerous and widespread group of phenolics in higher plants. Some of them inhibit intestinal motility *in vitro*. Quercetin produces relaxation in ileum contracted by KCl [28]. Apigenin and luteolin inhibited the contractions of isolated intestinum [29,30]. These substances have been reported to exhibit calcium antagonist and anticholinergic activities [31,32]. 

The medicinal properties of the genus *Helichrysum* are mainly attributed to the presence of flavanoids [33,34]. Our previous study showed that ethanol extracts of *H. plicatum* has high flavonoid heteroside content. Naringenin glycosides, apigenin-7-glucoside, quercetin- and kaempferol-3-glucoside were identified in notable concentration. Also, the amount of total phenolics varied from 101.1-266.9 mg GAE/g DW in different ethanolic extracts [35]. Based on this report, the spasmolytic activity of the ethanol extract of *H. plicatum* studied here could be attributed to flavanoids and other phenolic compounds. The key role of phenolic compounds as spasmolytic agents is emphasized in several reports. Our results are also in agreement with Mulatu [36] and Palacios-Espinosa [37] who have reported the spasmolytic activity of other plants species belonging to the same (Asteraceae) family. 

## 3. Experimental

### 3.1. Plant material

Flowers of *H. plicatum* were purchased from a commercial supplier (Agroherbal, Albania, batch number 29470605), in July 2007. Pulverization (sieve numbers according to European Pharmacopoeia 6.0. 2008) was carried out just before analysis. Flower heads of *H. plicatum* were extracted with aqueous ethanol (1:1) by triple percolation. Re-extraction were done by ethyl acetate-ethanol and then the extract was evaporated under vacuum at 60 °C to obtain a yellow-orange dried powder. The obtained extract was kept at 4 ^o^C. For experimental purposes the plant extract was first dissolved in ethanol (20% m/m), than diluted with distilled water to the appropriate concentration. Ethanol, at the same concentrations, had no effect on intestine contractility in the control experiments.

### 3.2. Solutions and drugs

The Tyrode solution used had following composition: 150 mM NaCl, 2.7 mM KCl, 2.00 mM MgCl_2_, 12 mM NaHCO_3_, 0.4 mM NaH_2_PO_4_, 1.8 mM CaCl_2_ and 5.5 mM glucose. The drugs used in biological tests were: acetylcholine chloride (Sigma, USA), histamine dihydrochloride (Sigma, USA), atropine sulphate (Sigma, USA) and papaverine hydrochloride (Merck, Germany). All drugs were dissolved in distilled water.

### 3.3. Animals

The research was conducted in accordance with the European Council Directive of November 24, 1986 (86/609/EEC). Male and female Wistar albino rats (200-250 g), 3-4 months of age, were used after a 24 h fasting with free access to water. Ileums were prepared and placed in 10 mL tissue baths containing Tyrode`s solution at 37 ºC and constantly aerated with a carbogen [38]. The preparations were equilibrated for 30 min. In the experiments 30 rats were sacrificed. The change of intestinal contractility was recorded using a transducer (TSZ-04-E, Experimetria Ltd, Budapest, Hungary), recorded and analyzed with a SPEL Advanced ISOSYS Data Acquisition System (Experimetria Ltd, Budapest, Hungary).

### 3.4. Experimental protocol

The tissues had been pretreated with the ethanol extract of *H. plicatum* (0.01–1 mg/mL). Papaverine (0.01–3 μg/mL) was used as control. In the second series of experiments, isolated intestinal segments were contracted by depolarization with 80 mM KCl. After tonic contractions were obtained, extract of *H. plicatum* (0.01–1 mg/mL) was cumulatively added at 15 min intervals. Papaverine (0.01–3 μg/mL) was added to the bath. Increasing concentrations of acetylcholine (5–1500 nM), histamine (1–300 nM) and barium chloride (3–900 μM) were added to the organ bath cumulatively to generate full concentration response curves. Then concentration response curves were obtained in the presence of the extract of *H. plicatum* (0.03–0.3 mg/mL) in the organ bath. 

### 3.5. Statistical analysis

Mean and standard error values were calculated for each group of results (n = 6 for each set of experiments) and significance of differences between the means were determined by the Student`s t-test. A probability value of p < 0.05 or less was noted as indicative of significance. An EC_50_ value (concentration of drugs causing half-maximal responses) was established by regression analysis.

## 4. Conclusions

The results obtained from the present study indicate that the ethanol extract of *Helichrysum plicatum* flowers showed a relaxant effect on isolated rat intestine. The extract of *H. plicatum* can inhibit spontaneous ileum contractions and contractions induced by acetylcholine, histamine, barium and potassium ions. The therapeutic effectiveness in the treatment of gastrointestinal disorders and use in traditional medicine of this plant could be due to its spasmolytic effect.
